# Phase Ib Study of Ulixertinib Plus Gemcitabine and Nab-Paclitaxel in Patients with Metastatic Pancreatic Adenocarcinoma

**DOI:** 10.1093/oncolo/oyac237

**Published:** 2022-11-25

**Authors:** Patrick M Grierson, Benjamin Tan, Katrina S Pedersen, Haeseong Park, Rama Suresh, Manik A Amin, Nikolaos A Trikalinos, Deborah Knoerzer, Brent Kreider, Anupama Reddy, Jingxia Liu, Channing J Der, Andrea Wang-Gillam, Kian-Huat Lim

**Affiliations:** Division of Medical Oncology, Department of Internal Medicine, Washington University, St. Louis, MO, USA; Division of Medical Oncology, Department of Internal Medicine, Washington University, St. Louis, MO, USA; Division of Medical Oncology, Department of Internal Medicine, Washington University, St. Louis, MO, USA; Division of Medical Oncology, Department of Internal Medicine, Washington University, St. Louis, MO, USA; Division of Medical Oncology, Department of Internal Medicine, Washington University, St. Louis, MO, USA; Section of Hematology/Oncology, Norris Cotton Cancer Center, Dartmouth-Hitchcock Medical Center, Lebanon, NH, USA; Division of Medical Oncology, Department of Internal Medicine, Washington University, St. Louis, MO, USA; BioMedValley Discoveries, Kansas City, MO, USA; BioMedValley Discoveries, Kansas City, MO, USA; BioMedValley Discoveries, Kansas City, MO, USA; Division of Public Health Sciences, Department of Surgery, Washington University, St. Louis, MO, USA; Department of Pharmacology, Lineberger Comprehensive Cancer Center, University of North Carolina at Chapel Hill, NC, USA; Division of Medical Oncology, Department of Internal Medicine, Washington University, St. Louis, MO, USA; Division of Medical Oncology, Department of Internal Medicine, Washington University, St. Louis, MO, USA

**Keywords:** ERK, ulixertinib, pancreatic cancer, gemcitabine

## Abstract

**Background:**

Ulixertinib is a novel oral ERK inhibitor that has shown promising single-agent activity in a phase I clinical trial that included patients with *RAS*-mutant cancers.

**Methods:**

We conducted a phase Ib trial combining ulixertinib with gemcitabine and nab-paclitaxel (GnP) for untreated metastatic pancreatic adenocarcinoma. The trial comprised a dose de-escalation part and a cohort expansion part at the recommended phase II dose (RP2D). Primary endpoint was to determine the RP2D of ulixertinib plus GnP and secondary endpoints were to assess toxicity and safety profile, biochemical and radiographic response, progression-free survival (PFS) and overall survival (OS).

**Results:**

Eighteen patients were enrolled. Ulixertinib 600 mg PO twice daily (BID) with GnP was initially administered but was de-escalated to 450 mg BID as RP2D early during dose expansion due to poor tolerability, which ultimately led to premature termination of the study. Common treatment-related adverse events (TRAEs) were anemia, thrombocytopenia, rash and diarrhea. For 5 response evaluable patients, one patient achieved a partial response and 2 patients achieved stable disease. For 15 patients who received the triplet, median PFS and OS were 5.46 and 12.23 months, respectively.

**Conclusion:**

Ulixertinib plus GnP had similar frequency of grade ≥3 TRAEs and potentially efficacy as GnP, however was complicated by a high rate of all-grade TRAEs (ClinicalTrials.gov Identifier: NCT02608229).

## Teaser

Ulixertinib has shown promising single-agent activity in a phase I clinical trial that included patients with RAS-mutant cancers. This phase Ib trial combined ulixertinib with gemcitabine and nab-paclitaxel for untreated metastatic pancreatic adenocarcinoma. Results are reported here.

**Lessons Learned**

- Ulixertinib plus gemcitabine and nab-paclitaxel (GnP) had similar grade ≥3 treatment-related adverse events as GnP; however, the triplet regimen is associated with poor overall tolerability, especially fatigue, nausea/vomiting, and diarrhea, leading to patient withdrawal from study.
- Ulixertinib plus gemcitabine and nab-paclitaxel (GnP) may have at least comparable efficacy as GnP but is complicated by high patient dropouts.
- Ulixertinib achieved on-target effect by downregulating an intra-tumoral *KRAS* gene signature based on RNA-sequencing (RNA-Seq) analysis. However, immunohistochemical staining to monitor phospho-ERK and the expression of the ERK phosphorylation substrate MYC were unable to confirm on-target effect.
- Treatment strategies to overcome adaptive mechanisms to ERK inhibition and with overall better tolerability are needed.

## Discussion

Pancreatic ductal adenocarcinoma (PDAC) is predicted to be the second leading cause of cancer death after 2030, and there are projected to be 62 210 new diagnoses and 49 830 deaths in the US in 2022.^1^. Combination chemotherapies including FOLFIRINOX and gemcitabine and nab-paclitaxel (GnP) remain the mainstay treatment for most patients, producing median OS of 11.1 and 8.5 months, respectively.^2,3^

Greater than 95% of PDAC carries the KRAS oncogene which results in a constitutive activation of the 3-tiered RAF-MEK-ERK mitogen-activated protein kinase (MAPK) cascade.^4-6^ However, targeting either RAF or MEK alone was unsuccessful in the clinic,^7,8^ in part due to loss of ERK-dependent negative feedback loops that drove rapid adaptive mechanisms that reactivate ERK kinases.^9,10^ Ulixertinib (BioMed Valley, Kansas City, USA) is a novel ATP-competitive ERK1/2-selective kinase inhibitor that has demonstrated activity in preclinical colon and gastric cancers and PDAC xenograft models.^11-13^ In a phase I study of advanced solid malignancies, ulixertinib showed activity with an overall response rate (ORR) of 14% at RP2D of 600 mg twice daily (BID) and a tolerable toxicity profile.^14^ On this basis, we conducted a phase Ib study of ulixertinib in combination with GnP for patients with untreated metastatic PDAC (NCT02608229).

**Figure 1.**
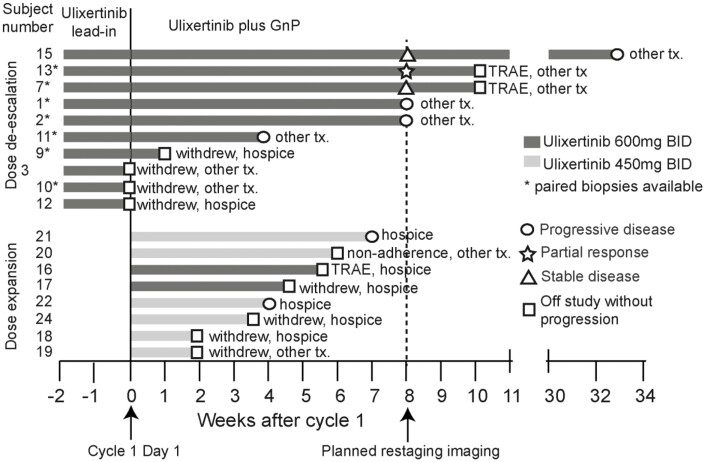
Treatment course of all 18 enrolled patients. 15 patients received ulixertinib plus GnP.

A total of 18 patients were enrolled into 2 parts: dose de-escalation and dose expansion. In dose de-escalation, 10 enrolled patients received ulixertinib monotherapy (600 mg BID) for 2 weeks. Seven patients continued on the triplet with ulixertinib 600 mg BID. In dose expansion, after 2 patients began treatment on triplet and withdrew. In response, ulixertinib was adjusted from 600mg BID to 450 mg BID as RP2D, and 6 more patients were enrolled. For 15 patients treated with the triplet, overall grade ≥ 3 TRAEs of the triplet were similar to standard GnP. One patient died from pneumonitis possibly related to gemcitabine. The high frequency of all-grade TRAE from the triplet, especially fatigue, nausea/vomiting and diarrhea, led to patient withdrawals, ultimately leading to premature study termination (Table 1). Only 5 patients received at least 2 cycles of treatment and were radiographically evaluable: 1 had PR, 2 had SD and 2 had PD (Fig. 1).

**Table 1.** Triplet regimen treatment related adverse events (TRAE) of enrolled patients (*N* = 15).

Adverse event	Grade 1/2, *n* (%)	Grade ≥3, *n* (%)	Any grade, *n* (%)
Constitutional			
Fatigue	8 (53)	0 (0)	8 (53)
Anorexia	3 (20)	0 (0)	3 (20)
Nausea/Vomiting	5 (33)	4 (27)	9 (60)
Hematologic			
Neutropenia	0 (0)	6 (40)	6 (40)
Thrombocytopenia	11 (73)	3 (20)	14 (93)
Anemia	11 (73)	3 (20)	14 (93)
Nervous system			
Peripheral sensory neuropathy	7 (47)	0 (0)	7 (47)
Pulmonary			
Pneumonitis	0 (0)	1 (grade 5; 6.7)	1 (6.7)
Gastrointestinal tract			
Intraabdominal infection	0 (0)	1 (6.7)	1 (6.7)
Constipation	1 (6.7)	0 (0)	1 (6.7)
Diarrhea	9 (60)	2 (13)	11 (73)
Liver			
AST increase	3 (20)	1 (6.7)	4 (27)
ALT increase	4 (27)	1 (6.7)	5 (33)
ALP increase	2 (13)	0 (0)	2 (13)
Kidney and electrolytes			
Elevated creatinine	1 (6.7)	0 (0)	1 (6.7)
Hyponatremia	0 (0)	1 (6.7)	1 (6.7)
Dermatologic			
Skin rash	12 (80)	2 (13)	14 (93)
Other			
Blurred vision	2 (13)	0 (0)	2 (13)

**Table:** 

Trial Information	
Disease	Pancreatic ductal adenocarcinoma
Stage of disease/treatment	IV
Prior therapy	None
Type of study	Phase Ib
Primary endpoint	Recommended phase II dose of ulixertinib in combination with Gemcitabine and nab-paclitaxel
Secondary endpoints	Safety and toxicity profile, biochemical and radiographic response, time to tumor progression (TTP), progression-free survival (PFS), and overall survival (OS) for patients treated with ulixertinib in combination with GnP
Investigator’s analysis	Active but too toxic as administered in this study. Correlative endpoints met but not powered to assess activity. Poorly tolerated/not feasible

## Additional Details of Endpoints or Study Design

### Materials and Methods

#### Study Design and Participants

This was a single institution, phase Ib, open-label study conducted at Washington University in St Louis, MO, USA to evaluate the safety and tolerability of ulixertinib plus GnP for patients with untreated metastatic PDAC under institutional review board approval (IRB# 201601098). The trial comprised a dose de-escalation portion and an expansion cohort at the RP2D. All patients provided written informed consent, and the study was conducted in accordance with the Declaration of Helsinki. Eligible patients were at least 18 years old and had histologically proven untreated metastatic PDAC. Inclusion criteria required ECOG < 1 with normal bone marrow and organ function (absolute neutrophil count >1500/μL, platelets >10 000/μL, hemoglobin >9 gm/dL, total bilirubin <ULN, AST, and ALT <2.5 × ULN, creatinine <1.5 × ULN or GFR >50 mL/minutes, cardiac left ventricular ejection fraction >50% and QTc < 470 ms. Exclusion criteria included CNS or brain metastases, significant ascites requiring therapeutic paracentesis or gastrointestinal conditions that could impair absorption of ulixertinib or HIV positivity.

#### Procedures

In the dose de-escalation phase, patients first received a 2-week lead-in of ulixertinib monotherapy according to a modified Fibonacci design with 3 decreasing dose levels (600 mg, 450 mg and 300 mg BID, at least one hour before food or 2 hours after food) to determine the RP2D. Patients experiencing excessive toxicity during the lead-in were replaced and were not to be considered evaluable for further toxicity assessment with combination treatment. Adverse events including severity and relationship to treatment were tabulated. Pre- and on-treatment tumor biopsies were performed during this period. After the 2-week ulixertinib lead-in, gemcitabine and nab-paclitaxel starting at 1000 mg/m^2^ and 125 mg/m^2^, respectively, on days 1, 8, 15 of a 28-day cycle were added. Dose-limiting toxicity (DLT) was defined as toxicity that occurred during the first cycle of the triplet regimen in the dose de-escalation phase that was possibly, probably or definitely related to ulixertinib. Hematologic DLT was defined as > grade 4 hematologic toxicity or grade 3 hematologic toxicity with complications (such as thrombocytopenia with bleeding). Non-hematologic DLTs included grade > 3 nausea, vomiting, or diarrhea lasting greater than 72 hours despite adequate treatment, but excluded grade >3 untreated nausea, vomiting, constipation, pain or rash. Non-hematologic DLTs also included a treatment interruption exceeding 14 days in cycle 1 due to ulixertinib related toxicity or any toxicity attributed to ulixertinib that resulted in a delay in gemcitabine and nab-paclitaxel for more than 4 weeks. For the dose expansion cohort, 25 patients were planned to be treated at the RP2D. Toxicity was graded per Common Terminology Criteria for Adverse Events v.4.0 (CTCAE v 4.0). Treatment response was radiographically assessed every 2 cycles and serum tumor marker (CA19-9) was measured every cycle. Treatment was continued until disease progression, unacceptable adverse events, or patient withdrawal of consent.

#### Outcomes

The primary endpoint was to determine the RP2D of ulixertinib in combination with GnP. The secondary endpoints included safety and toxicity profile, biochemical and radiographic response, progression-free survival (PFS), and overall survival (OS) for patients treated with ulixertinib in combination with GnP.

#### Correlative Studies

Imaging-guided biopsies were performed before and after 2 weeks of ulixertinib lead-in for patients in dose de-escalation phase. Biopsied samples were subjected to formalin-fixation and paraffin-embedment for immunohistochemistry and snap-frozen for RNA-Seq.

#### Statistical Analysis

Laboratory data measured on a continuous scale will be characterized by summary statistics (mean and standard deviation, SD). Alive patients were censored at the last follow-up. Progression-free survival (PFS) is defined as the months from the date of C1D1 treatment to progression or death. Alive patients without progression are censored at the death or last follow-up. Survival probabilities and progression-free probabilities at specific time points were calculated using Kaplan-Meier plot. SAS Version 9.4 (Cary, NC) was used to perform all statistical analyses.

#### Response Assessments

Treatment response was assessed according to the Response Evaluation Criteria in Solid Tumors version 1.1 (RECIST v1.1). TTP was defined as the time from treatment start (C1D1) to time of disease progression/withdrawal/end of study treatment. Overall survival (OS) was defined as the months from the date of C1D1 to death.

#### Immunostaining of Tumor Biopsies

The immunostaining procedures for the biopsy tissues were similar to those described previously.^15,16^ Briefly, the biopsy tissue blocks were sectioned at 5 μm thickness, deparafﬁnized with xylene, rehydrated, and antigen retrieved on-line using the BondMaxTM autostainer (Leica Microsystems, Bannockburn, IL). The slides were incubated with the primary antibodies using the following conditions: anti-pERK1/2 (Cell Signaling Technology, Cat# 4376, 1:175 for 60 minutes); anti-MYC (Abcam, Cat# ab32072, 1:75 for 60 minutes); and anti-p16INK4a (Abcam, Cat# ab54210, 1:4000 for 15 minutes). The antibody binding signals were detected using the Mach 3 Rabbit HRP Polymer Detection kit (Biocare Medical, Pacheco, CA). Slides were scanned using an Aperio Scanscope (Leica Microsystems) and visualized using the Aperio Image scope software (Leica Microsystems).

**Table:** 

Drug Information	
Generic/working name	nUlixertinib (BVD-523)
Company name	BioMed Valley Discoveries
Drug type	Small molecule reversible, ATP-competitive kinase inhibitor
Drug class	ERK1/2 inhibitor
Dose	600 mg or 450 mg
Unit	mg
Route	Oral
Schedule of administration	Twice daily

**Table:** 

Patient Characteristics	
Number of patients, male	13
Number of patients, female	5
Stage	IV
Age: median (range)	63 (37-78)
Number of prior systemic therapies	0
Performance status: ECOG	0: 6
	1: 12
	2: 0
	3: 0
	4: 0
Cancer types or histologic subtypes	Pancreatic ductal adenocarcinoma, 18

**Table:** 

Primary Assessment Method	
Number of patients screened	24
Number of patients enrolled	18
Number of patients evaluable for toxicity	18
Number of patients evaluated for efficacy	5 for radiographic assessment, 15 for PFS/OS
Evaluation method	RECIST 1.1; Tumor Marker
Response assessment, CR	0 (0%)
Response assessment, PR	1 (20%)
Response assessment, SD	2 (40%)
Response assessment, PD	2 (40%)
Median duration assessment, PFS	5.46 months (CI: 1.61-8.32)
Median duration assessment, OS	12.2 months (CI: 3.48-13.51)

## Outcome Notes

### Results

#### Enrollment

**Figure 2.**
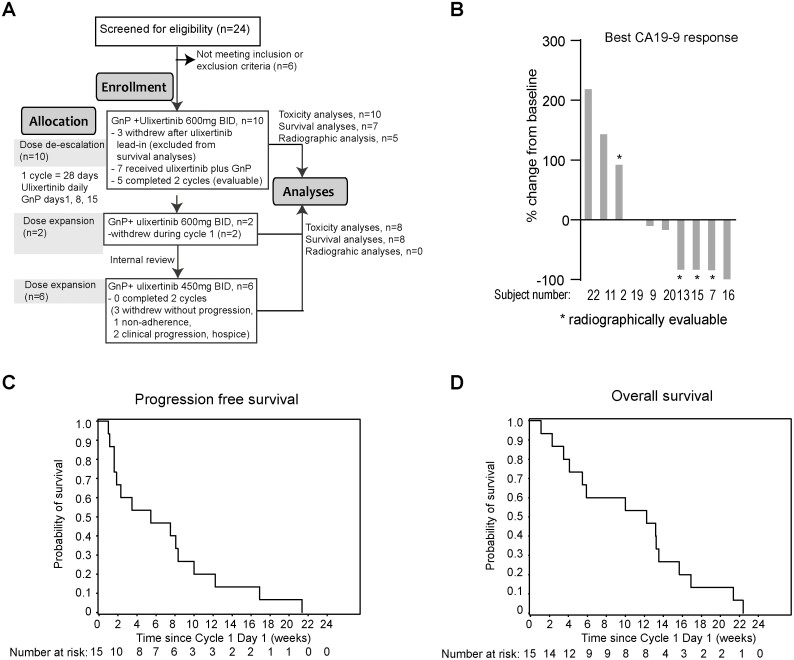
(**A**) Consort diagram showing enrollment of patients on this study. (**B**) CA19-9 percent change from baseline of 9 patients with available pre- and post-treatment CA19-9 values. Three other patients without elevated CA19-9 values were not included. (**C**) PFS and (**D**) OS of all 15 patients who started triplet therapy.

Between August 2016 to January 2019, a total of 24 patients were screened and 18 patients were enrolled and treated on study (Table 2). Of these, 10 patients were treated on dose de-escalation, starting from ulixertinib 600 mg BID. Of these 10 patients, 3 withdrew after the 2-week ulixertinib monotherapy lead-in as a result of fatigue attributed to disease progression and possibly to ulixertinib. Seven patients initiated on triplet therapy. However, prior to completion of cycle one, which was required for DLT assessment, one patient came off due to worsening disease-related symptoms (deemed to have clinical progression) and another patient decided to pursue hospice. Ulixertinib 600 mg BID in combination with GnP was determined as RP2D (Fig. 2A).

**Table 2.** Baseline characteristics of all enrolled patients.

Ulixertinib dose	Dose de-escalation	Dose expansion		All (*N* = 18)
	600 mg BID (*n* = 10)	600 mg BID (*n* = 2)	450 mg BID (*n* = 6)	
Age in years, median (range)	66 (56-74)	61 (37-78)		63 (37-78)
Sex				
Male	7	1	5	13
Female	3	1	1	5
Race				
Non-Hispanic/Caucasian	10	2	6	18
ECOG PS				
0	4	1	1	6
1	6	1	5	12
Evaluable for toxicities	10	2	6	18
Evaluable for survival	7	2	6	15

Dose expansion phase began in April 2018 and was halted after 2 patients were enrolled. One patient was hospitalized for anemia and pneumonitis attributed to gemcitabine during cycle 2 of the triplet combination, and later died. The other patient withdrew from the study due to treatment-related fatigue, nausea, and dehydration during cycle 1. Due to these concerns, after an internal review, RP2D of ulixertinib was adjusted to 450 mg BID in combination with GnP. Six additional patients were then treated. However, these patients were unable to complete 2 cycles of treatment due to clinical progression (*n* = 2) or intolerance to treatment leading to decision to withdraw (*n* = 3) and treatment non-adherence (*n* = 1). The study was subsequently closed by the sponsor due to poor tolerability. Of the 15 patients treated with the triplet regimen, 7 patients came off study prior to 2 cycles due to treatment intolerance (*n* = 5), adverse event (*n* = 1, grade 5 pneumonitis), non-adherence (*n* = 1) and clinical disease progression (*n* = 3). Therefore, 5 patients who completed 2 cycles of treatment were deemed evaluable for disease response (Fig. 1).

#### Safety

Treatment-related adverse events (TRAE) were documented for all 18 enrolled patients. The most common (observed in >15% of patients) grade ≥ 3 triplet regimen TRAEs included neutropenia (40%), nausea/ vomiting (27%), thrombocytopenia (20%), and anemia (20%, Table 1); these rates are similar to that reported for GnP. One patient developed pneumonitis and later died, determined to be possibly related to gemcitabine. Common all-grade TRAE for the triplet included fatigue (53%), nausea/vomiting (60%), diarrhea (73%), skin rash (93%), neutropenia (93%), and anemia (93%), which resulted in high patient withdrawal from the study. There was no grade ≥3 ulixertinib TRAEs observed in >15% of patients (Table 3). The most common ulixertinib TRAEs were predominantly grade 1/2 and consisted of rash (72%), diarrhea (44%), and fatigue (33%).

**Table 3.** Ulixertinib treatment related adverse events (TRAE) of enrolled patients (*N* = 18).

Adverse event *N* (%)	Grade 1/2	Grade 3/4	Any grade
Constitutional			
Fatigue	6	0	6 (33)
Anorexia	2	0	2 (11)
Nausea/Vomiting	0	2	2 (11)
Hematologic			
Neutropenia	0	2	2 (11)
Thrombocytopenia	4	1	5 (27)
Anemia	4	1	5 (27)
Nervous system			
Peripheral sensory neuropathy	0	0	0
Pulmonary			
Pneumonia	0	0	0
Pneumonitis	0	0	0
Gastrointestinal tract			
Constipation	1	0	1 (5)
Diarrhea	6	2	8 (44)
Liver			
AST increase	2	1	3 (17)
ALT increase	2	1	3 (17)
ALP increase	1	0	1 (5)
Hyperbilirubinemia	0	0	0
Kidney and electrolytes			
Elevated creatinine	1	0	1 (5)
Hyponatremia	0	1	1 (5)
Dermatologic			
Skin rash	11	2	13 (72)
Others			
Blurred vision	2	0	2 (11)

#### Efficacy

Of the 5 patients who finished 2 cycles of treatment and underwent planned restaging scans and therefore were radiographically evaluable (all of whom were treated in the 600 mg BID dose de-escalation phase), 2 had stable disease (SD), one had partial response (PR) and 2 had progressive disease (PD) (Fig. 1). Therefore, of these 5 evaluable patients, ORR was 20% (1 of 5) and disease control rate (DCR) was 60% (3 of 5), similar to that reported for GnP.^3^ For evaluation of biochemical response in 15 patients treated with triplet regimen, 3 had normal CA19-9 at baseline, and 3 withdrew before a post-treatment CA9-9 was obtained. Of the remaining 9 patients, 4 experienced a CA19-9 decrease by greater than 50%, yielding a CA19-9 biochemical response rate of 44% (Fig. 2B). Three patients with elevating CA19-9 had either clinical or radiographic progression. All patients who started treatment on triplet therapy were assessed for PFS and OS. The median PFS (mPFS) was 5.46 months (95% CI: 1.61-8.32, Fig. 2C), and median OS (mOS) was 12.23 months (95% CI: 3.48-13.51, Fig. 2D).

## Result of Pharmacodynamic Analysis

**Figure 3.**
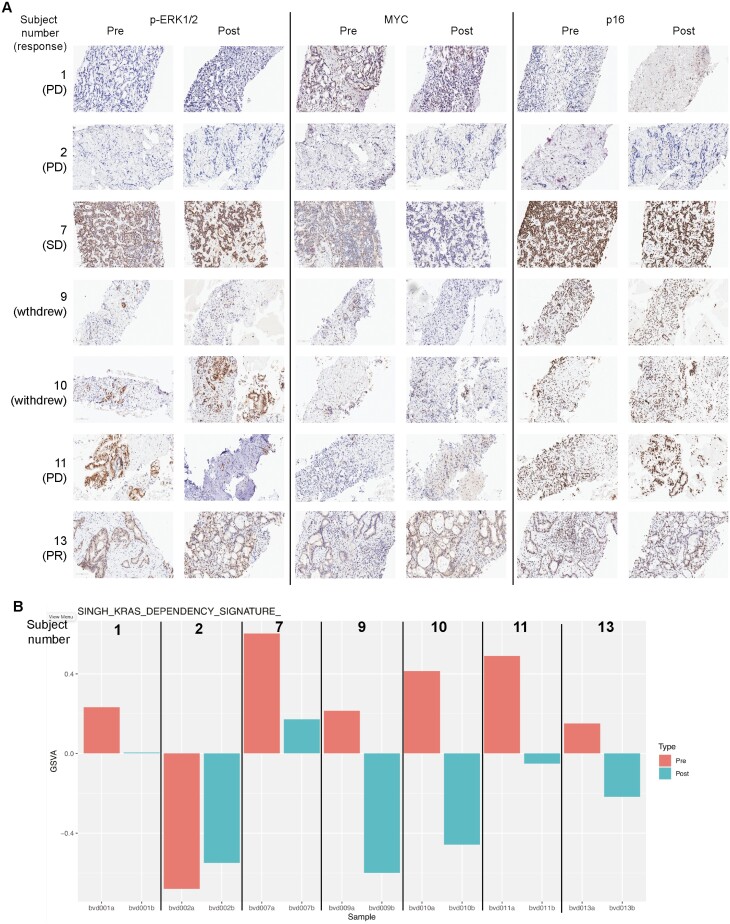
(**A**) IHC staining and (**B**) bulk RNaseq of 7 paired biopsy specimens treated with ulixertinib 600 mg BID lead-in for 2 weeks.

Based on published preclinical data in PDAC cell lines and xenograft models, ulixertinib should downregulate phosphorylated and activated ERK1/2 and p16 and MYC protein expression.^12,13^ MYC is phosphorylated and stabilized by ERK phosphorylation. We performed immunohistochemical analyses on these markers on 7 paired biopsies from patients treated with 2 weeks of ulixertinib 600 mg BID during lead-in period. Of these limited number of samples, we were unable to detect noticeable changes in staining intensity of these markers before and after ulixertinib treatment, and there seemed to be no correlation between basal staining intensity of these markers with treatment response (Fig. 3A). We then resorted to bulk RNA-Seq analysis, which we expected would provide a more sensitive assay for detection of the on-target effect of ulixertinib. Indeed, 6 of 7 ulixertinib-treated tumor samples exhibited downregulation of KRAS-dependence signature as defined by Singh et al^17^ (Fig. 3B). The KRAS-dependence signature consists of several target genes including SYK, TGFA, TMEM45B, ITGB6, ADAM8, FAM83A, LAMA3, FGFBP1, SH2D3A, LAMC2, RDHE2, DAPP1, LAD1, CLDN7, AREG, AMPD3, MST1R, MAOA, PRS22, and SEMA4B, which were identified from functional studies between KRAS-dependent and independent cell lines and found to be upregulated and essential in cells that critically depend on oncogenic KRAS for survival.^17^ Therefore, ulixertinib at 600 mg BID was capable of suppressing the expression of multiple KRAS-dependent target genes.

## Assessment, Analysis, and Discussion

**Table:** 

Completion	Study terminated prior to completion
Investigator’s assessment	Active but too toxic as administered in this study. Correlative endpoints met but not powered to assess activity. Poorly tolerated/not feasible

Despite the high frequency of KRAS mutations in PDAC, and the well-validated role for KRAS in supporting PDAC growth, until recently, direct therapeutic targeting of KRAS has not been a clinical option. Given the ability of activated RAF to phenocopy mutant KRAS and drive full development of invasive and metastatic cancer,^18^ targeting this key KRAS effector signaling pathway is considered an attractive option. The ERK1/2 kinases have recently emerged as a promising therapeutic target as they represent the final step in the 3-tiered RAF-MEK-ERK MAPK cascade and consequently, less vulnerable to pathway reactivation resistance mechanisms.^19^ This is the first study that combines an ERK inhibitor with standard GnP for PDAC patients. Overall, GnP plus ulixertinib led to similar frequencies of grade ≥3 TRAE and similar measures of efficacy (ORR, DCR, PFS, OS) compared with that reported for GnP; however, we observed a high rate of withdrawal from patients due to TRAEs especially fatigue, nausea/vomiting and diarrhea.

In a phase I study, ulixertinib monotherapy at 600 mg BID or above achieved a PR of 17% in patients with refractory NRAS or BRAF-mutant solid tumors including melanoma, gall bladder, glioblastoma multiforme, head and neck, and small-bowel cancer.^14^ Here we observed a similar spectrum of treatment-related side effects attributed to ulixertinib including rash, diarrhea, and fatigue which were predominantly low grade. One patient was admitted for anemia and pneumonitis attributed to gemcitabine, and later succumbed to respiratory failure.

Assessment of preliminary efficacy in our study was limited by overall poor treatment tolerance and high dropout rates before planned radiographic assessment. Despite this shortcoming, biochemical response (>50% decline) was observed in 44% of patients, which to some degree reflected the efficacy and strongly suggests the need to address the adaptive resistance mechanisms. For instance, ulixertinib treatment resulted in compensatory upregulation of protective autophagy and activation of the upstream HER family of receptor tyrosine kinases.^13,20^ Therefore, further development of ERK inhibitors in PDAC will require synergistic combinations that overcome these mechanisms and potentially allow each agent to be administered at a lower dose to improve tolerance. Analysis of tumor samples showed ulixertinib treatment resulted in downregulated KRAS pathway signature, suggestive of on-target effect, although we were unable to detect definitive changes in pERK and MYC IHC expression in our small number of samples. Whether ulixertinib administered at lower dose is able to sufficiently downregulate KRAS signaling to disrupt KRAS oncogenic function remains to be determined. In addition, the ideal therapeutic combinations should exhibit significant efficacy such as sustained tumor regression or sustained growth arrest in robust preclinical models such as the patient-derived xenograft models.

In conclusion, ERK kinases remain as important therapeutic target in PDAC but our study showed that ulixertinib in combination with GnP did not improve measures of efficacy, largely due to poor tolerability of this triplet. Ulixertinib at 600 mg achieved intratumoral on-target effect by lowering KRAS signatures. Future success in targeting ERK will require rational therapeutic combinations with robust preclinical efficacy and manageable side effects.

## Data Availability

The data underlying this article will be shared on reasonable request to the corresponding author. Bulk RNAseq data from biopsied tumors were deposited in GEO (Accession number GSE213797).
